# Psychological Distress and Trust in University Management Among International Students During the COVID-19 Pandemic

**DOI:** 10.3389/fpsyg.2021.679661

**Published:** 2021-06-18

**Authors:** Karamat Khan, Yanyan Li, Sheng Liu, Chuntao Li

**Affiliations:** ^1^School of Economics, Henan University, Kaifeng, China; ^2^School of Education, Henan University, Kaifeng, China; ^3^School of Business, Anhui University, Hefei, China; ^4^School of Finance, Zhongnan University of Economics and Law, Wuhan, China; ^5^School of International Education, Huanghuai University, Zhumadian, China

**Keywords:** COVID-19, university management, trust, anxiety, self-quarantine

## Abstract

Since the end of 2019, the outbreak of the COVID-19 pandemic has engendered widespread fear and anxiety across China. Nearly half a million international students pursuing their studies in Chinese universities have also been exposed to the psychological distress triggered by the unfolding crisis. In addition to government and medical institutions' efforts, universities have also endeavored to mitigate concerns among these students under quarantine on campus by providing reliable information as well as medical, monetary, and emotional support. In this study, international students' trust in university management teams and its role in remediating their anxieties were evaluated using an online survey conducted after 10 days of the lockdown of Wuhan, China. The empirical analysis incorporates quantitative data from 180 international students. Ordinary least squares regression and probit regression were used in the analysis with the non-robust and robust models. The study found students' perception of trust in university management to be negatively associated with their anxiety levels. Additionally, having trust in university management was found to positively influence students' commitment to the self-quarantine guidelines. These results reinforce the important role of universities and their relationship with international students during public health emergencies.

## Introduction

The novel coronavirus (COVID-19) was first identified in Wuhan, Hubei province, China, at the end of 2019 and spread rapidly from one location to another, causing panic worldwide. Around 132 million people have been infected worldwide by the COVID-19 pandemic, with over 2.86 million fatalities reported by the end of March 2021 (World Health Organization, 2021). The effects of the COVID-19 pandemic have been significant, reaching beyond national health care sectors and into social, political, cultural, and economic domains (Aucejo et al., 2020; Bartik et al., 2020; Cao et al., 2020; Flesia et al., 2020; Khan et al., 2020).

A wide range of psychological issues, such as anxiety, loss, grief, suspicion, and fear, have been experienced by individuals, families, groups, and communities during the crisis (AlAteeq et al., 2020; Asmundson and Taylor, 2020; Liu et al., 2020; Xiang et al., 2020). Some groups of people would appear to be more vulnerable than others in terms of susceptibility to the disease as well as to other associated challenges emerging from the pandemic (Aylie et al., 2020; Flesia et al., 2020; Sheroun et al., 2020). Students are one such group that has attracted extensive attention from society and academia alike (Cao et al., 2020; Amendola et al., 2021; Conrad et al., 2021; Wang et al., 2021; Zeng et al., 2021). For instance, the pandemic forced many universities to close their campuses and shift to online learning. COVID-19-related psychological stress combined with the sudden changes in learning methods posed significant problems for students, with negative consequences for their mental health (Cao et al., 2020; Collins, 2021; Wang et al., 2021; Zeng et al., 2021).

Compared to local students, international students pursuing their studies abroad face more complicated and unique challenges (Chen et al., 2020). Even under normal circumstances, international students are more vulnerable to psychological distress due to difficulties accessing medical care and are less motivated to seek out psychological services than their domestic peers (Alharbi and Smith, 2018; Brunsting et al., 2018). The vulnerability of international students intensifies during a crisis like COVID-19 owing to their lack of access to public resources, financial constraints, cultural or language barriers, inability to access reliable information, and the absence of the basic necessities of life (Park and Lee, 2016; Chen et al., 2020; Lee et al., 2021).

Moreover, some campuses were closed without recognition of the fact that many international students may not have a place to live outside of such campuses, or would not be able to access a secure return to their home countries as a result of closed borders, limited international flights, and the possibility of exposure to COVID-19 during travel (Ma et al., 2020; Conrad et al., 2021; Gewirtz O'Brien et al., 2021; Mok et al., 2021). Those who remained in their host countries were faced with unmet psychological needs concerning relatedness as a result of their physical separation from loved ones and a loss of social support in the local culture—not to mention the psychosocial issues involved with wider society's response to COVID-19 (Chen et al., 2020; Fakhar-e-Alam Kulyar et al., 2020; Conrad et al., 2021).

In general, many studies have investigated students' mental health and coping strategies amid the COVID-19 pandemic (Aucejo et al., 2020; Aylie et al., 2020; Cao et al., 2020; Amendola et al., 2021; Conrad et al., 2021; Wang et al., 2021; Zeng et al., 2021; Zhang et al., 2021). Our review suggests that international students, as a minority group on campus, face greater impediments to maintaining their mental health during the COVID-19 pandemic, and may require more attention because of their unique challenges and stressors. However, prior studies have often omitted a particular focus on international students' needs, or have addressed them as being the same as for local students. Consequently, there is an opportunity to add to this under-researched area by investigating the impact of the pandemic on the mental health of international students and the coping strategies that could help to reduce their anxieties during the crisis (Amoah and Mok, 2020; Chen et al., 2020; Amendola et al., 2021).

This study is specifically focused on international students who are studying in China. Its principal hypothesis is that international students with higher trust in their university's management will experience less anxiety during the COVID-19 pandemic crisis. According to Stolle (2001), “trust” refers to a sense of anticipated support provided mostly unconditionally from something being trusted. In the present study's context, we define trust as a student's confidence in their university's measures and support structures in place to prevent them from contracting the disease and experiencing psychological distress. For example, many universities restricted students' movement, started online classes, and provided food, medicine, and other groceries on their doorsteps free of cost or at subsidized prices during the crisis. “University management” here refers to a university's management at any level, but also specifically the international school of the university that is responsible for the affairs of the international students attending the university.

Further to our principal hypothesis, we also postulate that trust in university management will positively affect students' tendency to accept self-quarantine behavior. Correspondingly, our study's findings will also help reveal the extent to which international students practice the preventive measures recommended by the Chinese Center for Disease Control and Prevention (2020) and the World Health Organization (2020a). Thus, the results of this study provide meaningful evidence that can help governmental and educational institutions take effective steps to support such vulnerable populations in a pandemic situation, now and in the future.

## Materials and Methods

### Participants and Procedures

The study aimed to explore the impact of trust in university management on psychological distress among international students and their self-quarantine behavior during the COVID-19 pandemic. For this purpose, an online cross-sectional study (see Supplementary Material for the questionnaire) was undertaken from February 3 to 14, 2020—precisely 10 days after the Wuhan lockdown on January 23, 2020. We approached participants through official WeChat groups that had already been developed by the universities for their international students. During the online investigation, international students were quarantined in their campus dormitories/off-campus residences, due to a government lockdown policy. The participants were recruited using a non-probability sampling technique (a combination of purposive and convenience sampling techniques). We collected 180 valid responses from international students located in several places in Hubei, but from Wuhan in particular. Although a portion of the participants was not actually in Wuhan, the epicenter of the outbreak, they would have been closely monitoring social media related to the pandemic.

### Measures

#### Trust

We used a 10-item scale to assess international students' trust in the university's management team (Warner-Søderholm et al., 2018). The scale was adapted to our study context, and included two items from each of the five subdimensions of the trust construct: benevolence, integrity, competence, identification, and concern (e.g., “University management really does care about the well-being of international students”). The items were rated on a 5-point scale ranging from 1 (*strongly disagree*) to 5 (*strongly agree*). If a reverse-coded question was used, it was converted into the same direction at the data entry stage. The scale had an alpha reliability coefficient of 0.949.

#### Anxiety

The Clinical Anxiety Scale (Westhuis and Thyer, 1989) was adapted to measure the anxiety levels of the respondents on a 5-point Likert scale (Quah and Hin-Peng, 2004). Our instrument consisted of 10 items in relation to which respondents indicated their level of agreement with the statements using a rating from 1 (*strongly disagree*) to 5 (*strongly agree*). For example, we asked participants, “Thinking of how you feel these days, would you say ‘I feel calm'?” Negative item scores were reversed, so lower total scores indicated higher anxiety. The scale had an alpha reliability coefficient of 0.886.

#### Acceptance of Self-Quarantine

Following Quah and Hin-Peng (2004), we asked the respondents if they would be willing to self-quarantine themselves if they had had non-close contact with a COVID-19-infected person. Responses were given on a 5-point scale ranging from 1 (*strongly disagree*) to 5 (*strongly agree*). We created a dichotomous variable based on the average score on the item (*M* = 3.78; *SD* = 1.19). The variable took a value of 1 if the respondent showed higher acceptance for self-quarantine (i.e., score >4), and 0 otherwise.

### Control Variables

We controlled for several variables that might affect the dependent variables, to ensure a rigorous test of the primary hypothesis of this study.

#### Essential Knowledge of COVID-19

Participants' essential knowledge about the disease may affect their anxiety levels and attitude to accepting self-quarantine. We used five questions to test each participant's knowledge of the COVID-19 disease. Responses were scored 0 (*incorrect*) or 1 (*correct*); a composite index indicated the number of correct answers, from none correct (0) to all five correct (5). We created a dichotomous variable based on the average score for knowledge of COVID-19 (*M* = 2.93; *SD* = 1.10). The variable took a value of 1 if the respondent had a greater knowledge of the disease (score >3), and 0 otherwise.

#### Self-Health Perception

Individual self-health perception may also influence a person's anxiety levels and tendency to accept self-quarantine. We asked the participants if they had had, in the preceding 2 weeks, any of the six physical health symptoms (e.g., flu symptoms, high temperature, sore throat) that are associated with the COVID-19 disease (Chinese Center for Disease Control and Prevention, 2020; World Health Organization, 2020b). We created a dichotomous variable that took a value of 1 if the participant reported at least one physical health symptom during the previous 2 weeks, and 0 otherwise.

#### Demographic Characteristics

We also collected data on the demographic characteristics of the participants. Participants who were in Wuhan, male, married, and belonged to the age category of 30–39 years old were given a score of 1, while 0 was assigned for participants who were from outside Wuhan, female, unmarried, and in the age category of 20–29. According to a recent report, 77% of international students in China are originally from Asia or Africa (Ministry of Education PRC, 2019). Therefore, we constructed a variable consisting of three ethnic groups, distinguishing between participants of Asian origin, of African origin, and of other origins. Finally, information pertaining to the current educational levels of respondents to the survey was also collected that delineated whether students were studying Ph.D., master's, or undergraduate programs in any Chinese university.

#### Preventive Measures

The Chinese Center for Disease Control and the World Health Organization have recommended many preventive measures for the general public to adopt that could reduce the risks of transmitting or contracting COVID-19 (Chinese Center for Disease Control and Prevention, 2020; World Health Organization, 2020a). However, standard data collection surveys rarely include information about such preventive measures being adopted by the public during a significant outbreak. Therefore, in our study, we asked the international students to report the extent to which they had followed the preventive measures (e.g., “Over the past 2 weeks, I have washed my hands with water and soap before and after I leave my home/room/dormitory”).

## Results

### Descriptive Statistics

Means (*M*s), standard deviations (*SD*s), and frequencies are reported for our study's variables in Table 1. As shown, international students reported higher levels of trust in the university management (*M* = 3.997, *SD* = 0.829) and acceptance of self-quarantine (*M* = 3.783, *SD* = 1.188). The mean score for anxiety was 2.76 (*SD* = 0.790). The mean score pertaining to knowledge of COVID-19 was 2.928 (*SD* = 1.104) out of 5. Thus, the study's participants were found to possess approximately 58.56% of essential knowledge about COVID-19. Participants reported that they were practicing nearly all (*M* = 9.856, *SD* = 1.354) of the COVID-19 infection prevention measures recommended by the World Health Organization and the Chinese Center for Disease Control and Prevention. A total of 30 (16.67%) students reported that they had at least one of the COVID-19 symptoms.

**Table 1:** Descriptive statistics.

**Variables**	**M ± SD**	**Frequency (%)**
Anxiety	2.766 ± 0.790	
Trust	3.997 ± 0.829	
Self-quarantine	3.783 ± 1.188	
Knowledge	2.928 ± 1.104	
Preventive measures	9.856 ± 1.354	
Self-health perception		
Reported one symptom		30 (16.67%)
No symptom		150 (83.33%)
Location		
Wuhan		82 (45.56%)
Other		98 (54.44%)
Gender		
Male		119 (66.11%)
Female		61 (33.89%)
Age (range)		
20–29		122 (67.78%)
30–39		58 (32.22%)
Marital status		
Married		43 (23.89%)
Unmarried		137 (76.11%)
Education		
Ph.D.		90 (50%)
Master's degree		66 (36.67%)
Undergraduate		24 (13.33%)
Ethnic group		
Asian origin		123 (68.33%)
African origin		31 (17.22%)
Other origin		26 (14.25%)

*M, mean; SD, standard deviation*.

The demographic data collected show that participants from Wuhan were comparable in numbers to those from other locations (Wuhan = 45.56%, Other Cities = 54.44%). Further, 66.11% of participants were men (vs. 33.89% female), married 23.89% (vs. 76.11% unmarried), and 67.78% belonged to the age category of 20–29 (vs. 32.22% over the age of 29). Table 1 also details that 50% of the study's participants were pursuing their Ph.D. studies in China, 36.7% their master's degrees, and 13.3% were undergraduate students. Finally, the data show that 68.3% of respondents were of Asian origin, 17.2% from Africa, and the remainder were from other ethnic groups.

### Measures Taken for the Prevention of COVID-19

**Figure 1:**
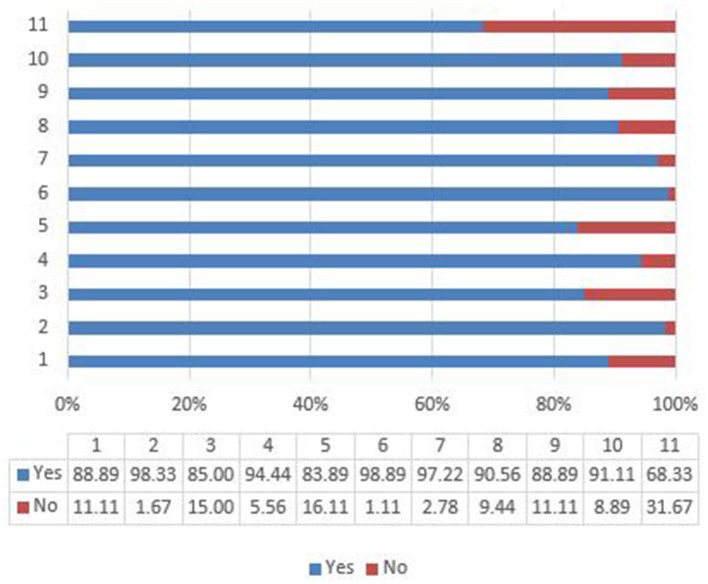
Preventive measures.

Figure 1 shows the respective proportions of respondents who started practicing each of the 11 measures recommended by the Chinese Center for Disease Control and Prevention and the World Health Organization for reducing the risks of contracting and transmitting COVID-19, directed against the two main modes of transmission (i.e., person-to-person droplet spread and fomites). Table 2 lists the preventive measures adopted by the participants. Most reported that they favored staying at home or in their room/dormitory during the outbreak (89%), washing their hands with soap before and after leaving home (98%), avoiding touching their face with their hands (85%), avoiding contact with people who have COVID-19 symptoms (94%), and using sanitizer to wash their hands when outside (84%). The participants also reported that they wore a mask (99%) when going outside, which is an important measure with which to reduce transmission of COVID-19 through fomites. Further, they avoided crowded places, visiting hospitals, and public transportation (97, 91, and 89%, respectively), and ate healthy food and took part in exercise (91 and 68%, respectively) in order to try to improve their immune systems.

**Table 2:** Preventive measures.

1	If possible, I did not leave home/room/dormitory.
2	I washed my hands with water and soap before and after I left home.
3	I didn't touch my eyes, nose, and/or mouth with hands I had not washed.
4	I avoided contact with people who had fever or respiratory symptoms.
5	I washed my hands with hand sanitizers when I was outside.
6	I wore a mask when I went outside.
7	I avoided crowded places.
8	I went to the hospital much less.
9	I avoided using public transportation.
10	I ate food that would strengthen my immune system.
11	I exercised to strengthen my immune system.

### Trust in University Management, Psychological Distress, and Self-Quarantine

This study explored the impact of trust in university management on psychological distress and self-quarantine behavior among international students during the COVID-19 pandemic. We used the ordinary least squares method for the continuous dependent variable, while the probit regression approach was employed for the binary dependent variable. For the probit regressions, we also report the marginal effects (MEs). We first estimated a non-robust model without adjusting for heteroscedasticity. This served as our baseline specification model. However, heteroscedasticity is considered a common problem with cross-sectional data. To reduce the influence of the heteroscedasticity of the variables that may exist in the regression models on the significance of the coefficients, a robust estimator of standard errors is used in regression analysis. We also checked for multicollinearity and normal distribution. These statistics were found to be in the acceptable range using different tests suggested by historical research. Data were analyzed with Stata (version 15).

We first examined the impact of trust in university management on international student's anxiety levels. In column 1 of Table 3, we present our baseline specification model. These results show that trust has a negative impact on anxiety but is weakly significant from a statistical viewpoint (β = −0.139, *p* < 0.10). However, the statistical significance strengthens in column 2 of Table 3, when we use heteroscedasticity robust standard errors (β = −0.139, *p* < 0.05). Overall, we find support for our hypothesis that international students' perception of trust in university management may decrease their anxiety levels. Moreover, we find that a participant's self-health perception is positively associated with their anxiety level. These results are statistically significant in both the baseline and robust models (β = −0.336, *p* < 0.05). With regard to other control variables, we did not find a significant impact on anxiety levels.

**Table 3:** Main results.

**Variables**	**Anxiety**	**Anxiety**	**Self-quarantine**		**Self-quarantine**	
	**(1)**	**(2)**	**(3)**	**(4)**	**(5)**	**(6)**
	***SE***	**Robust *SE***	***SE***	**ME**	**Robust *SE***	**ME**
Trust	−0.139^*^	−0.139^**^	0.326^**^	0.101^**^	0.326^**^	0.101^**^
	(0.0744)	(0.0685)	(0.147)	(0.0437)	(0.147)	(0.0444)
Knowledge	−0.0320	−0.0320	0.626^***^	0.194^***^	0.626^***^	0.194^***^
	(0.132)	(0.140)	(0.237)	(0.0693)	(0.240)	(0.0705)
Self-health	0.366^**^	0.366^**^	0.362	0.112	0.362	0.112
	(0.159)	(0.153)	(0.283)	(0.0865)	(0.274)	(0.0838)
Location	0.140	0.140	−0.0619	−0.0191	−0.0619	−0.0191
	(0.127)	(0.130)	(0.230)	(0.0710)	(0.218)	(0.0674)
Gender	0.0730	0.0730	0.537^**^	0.166^**^	0.537^**^	0.166^**^
	(0.125)	(0.123)	(0.234)	(0.0695)	(0.237)	(0.0697)
Marital status	0.208	0.208	0.692^**^	0.214^**^	0.692^**^	0.214^**^
	(0.156)	(0.156)	(0.286)	(0.0839)	(0.277)	(0.0840)
Age	−0.0594	−0.0594	−0.235	−0.0727	−0.235	−0.0727
	(0.153)	(0.149)	(0.284)	(0.0873)	(0.265)	(0.0817)
Education						
Ph.D.	0.197	0.197	−0.0111	−0.00343	−0.0111	−0.00343
	(0.198)	(0.193)	(0.363)	(0.112)	(0.354)	(0.110)
Master's	0.0795	0.0795	0.384	0.119	0.384	0.119
degree	(0.194)	(0.190)	(0.357)	(0.109)	(0.344)	(0.106)
Ethnic Group						
African origin	−0.209	−0.209	−0.950^**^	−0.294^**^	−0.950^**^	−0.294^**^
	(0.222)	(0.232)	(0.428)	(0.127)	(0.410)	(0.122)
Asian origin	−0.105	−0.105	−0.353	−0.109	−0.353	−0.109
	(0.179)	(0.186)	(0.317)	(0.0971)	(0.301)	(0.0926)
Constant	3.107^***^	3.107^***^	−2.277^***^		−2.277^***^	
	(0.371)	(0.344)	(0.729)		(0.716)	
Observations	180	180	180		180	
*R*^2^/Pseudo	0.095	0.095	0.1174		0.1174	

*SE, standard error; ME, marginal effect*.

*** *p < 0.01*;

** *p < 0.05*;

* *p < 0.10*.

Next, we investigated the influence of trust in university management on self-quarantine behavior of international students. We estimated a probit model since the variable we want to explain takes only two values. Initially, we estimated a non-robust model, and then we used heteroscedastic-robust standard errors to increase the robustness of our results. In general, we do not interpret the coefficients of the probit regression but rather the MEs. Marginal effects tell us that how much the (conditional) probability of the outcome variable changes with the change of the regressor, holding all other regressors constant. Accordingly, we report the MEs at the sample mean values of the regressors in columns 4 and 6 of Table 3. We find a significant and positive influence of trust in university management on self-quarantine behavior, not just in the non-robust model but also when we use heteroscedastic-robust standard errors (ME = 0.110, *p* < 0.05).

We also find that individuals with essential knowledge of COVID-19 disease are more likely to accept self-quarantine (ME = 0.194, *p* < 0.01). The results are consistent in both the baseline and robust models. Self-health perception is found to be insignificant both in a non-robust and a robust model. The results in relation to the demographic data show that male and married individuals have a higher probability of accepting self-quarantine than unmarried and female individuals (ME = 0.166, *p* < 0.05; ME = 0.214, *p* < 0.05). As for as ethnic group is considered, we find that individuals of African origin are less likely to exhibit self-quarantine behavior (ME = −0.294, *p* < 0.05). Finally, we do not find any significant relationships on the bases of location, age, and education.

## Discussion

The current study presents one of the first empirical investigations into the associations between international students' trust in university management, mental health, and acceptance of self-quarantine behavior. The findings of this study suggest that trust in university management is negatively associated with students' anxiety levels and can also positively influence students' willingness to comply with self-quarantine guidelines during the pandemic crisis.

People require advice from a trusted source on how to act in crisis situations (Yang and Cho, 2017; Ma et al., 2020). Governmental and public agencies are ideal sources of reliable information and immediate support in such contexts because citizens place a high premium on national administration. Therefore, citizens' acceptance of policies and practices are highly dependent on trust in these institutions (Yang and Cho, 2017). On the other hand, international students, because of their immigrant status and lack of familiarity with the local health care system, are more reliant on universities for emotional support and guidance (Amoah and Mok, 2020; Chen et al., 2020; Conrad et al., 2021). Anxiety, according to control/alienation theory, results from one's inability to control stress. Therefore, to compensate for one's inability, individuals seek others' help to gain control over stress (Sherman, 1987; Mirowsky and Ross, 1989; Ross and Mirowsky, 1989). Conceivably, expecting to obtain any sort of support from the university, or having trust in university management, may be one of the means available for international students to be able to control their anxiety.

The study's results also suggest that individual self-health perception is positively associated with anxiety. Furthermore, we found that male and married individuals are more likely to comply with quarantine guidelines than female, unmarried, or students of African origin. This finding is in line with those presented in the prior literature in which researchers showed that demographic characteristics may predict self-isolation behavior (Commodari and La Rosa, 2020; Nkire et al., 2021). Our study also found that essential knowledge about the disease is positively associated with self-quarantine. This finding is consistent with a recent study that showed quarantined persons had a greater knowledge of the disease and behavioral compliance toward quarantine measures (Yan et al., 2021). Finally, a significant proportion of the participants in our study were found to follow the preventive measures recommended by the World Health Organization and the Chinese Center for Disease Control and Prevention to reduce exposure to the disease and its subsequent transmission.

The findings of this study offer useful policy insights for higher education institutions across different parts of the world, specifically where these institutions are mainly dependent on international students as one of their primary funding sources or incomes. To prepare successfully for the unpredictable future that lies ahead in terms of the internationalization of education, universities must expand the current support system and help students in protecting themselves and in mentally dealing with this pandemic. We recommend that the hosting institutions, in collaboration with other concerned bodies, should develop innovative strategies to improve the psychological well-being of the students as well as expand the existing student counseling facilities.

The study was limited by its cross-sectional survey, small sample size, and convenience sampling technique. Future research should enroll a greater number of respondents using random sampling techniques. We also recognize that this study's sample was limited to only international students in China. Further research could tap the scope of the generality of this study in other settings. Despite these limitations, though, our study provides the first empirical evidence suggesting that building a trust-based relationship may improve international students' well-being under public health emergencies.

## Data Availability Statement

The original data presented in the study are included in the article/Supplementary Material, further inquiries can be directed to the corresponding author/s.

## Ethics Statement

The studies involving human participants were reviewed and approved by Research Ethics Committee of Zhongnan University of Economics and Law. The patients/participants provided their written informed consent to participate in this study.

## Author Contributions

All authors contributed to the article and approved the submitted version.

## Conflict of Interest

The authors declare that the research was conducted in the absence of any commercial or financial relationships that could be construed as a potential conflict of interest.

## References

[B1] AlAteeq D. A. Aljhani S. AlEesa D. (2020). Perceived stress among students in virtual classrooms during the COVID-19 outbreak in KSA. J. Taibah Univ. Med. Sci. 15, 398–403. 10.1016/j.jtumed.2020.07.004 32837508 PMC7395822

[B2] Alharbi E. S. Smith A. P. (2018). Review of the literature on stress and wellbeing of international students in English-speaking countries. Int. Educ. Stud. 11, 22–44. 10.5539/ies.v11n6p22

[B3] Amendola S. von Wyl A. Volken T. Zysset A. Huber M. Dratva J. (2021). A longitudinal study on generalized anxiety among university students during the first wave of the COVID-19 pandemic in Switzerland. Front. Psychol. 12:643171. 10.3389/fpsyg.2021.643171 33776867 PMC7990874

[B4] Amoah P. A. Mok K. H. (2020). The COVID-19 Pandemic and Internationalisation of Higher Education: International Students' Knowledge, Experiences, and Wellbeing. Emerald Blog. Available online at: https://www. emeraldgrouppublishing.com/opinion-and-blog/covid-19-pandemic-and-inte rnationalisation-higher-education-international-students (accessed May 05, 2021).

[B5] Asmundson G. J. G. Taylor S. (2020). How health anxiety influences responses to viral outbreaks like COVID-19: what all decision-makers, health authorities, and health care professionals need to know. J. Anxiety Disord. 71:102211. 10.1016/j.janxdis.2020.102211 32179380 PMC7271220

[B6] Aucejo E. M. French J. Ugalde Araya M. P. Zafar B. (2020). The impact of COVID-19 on student experiences and expectations: evidence from a survey. J. Public Econ. 191:104271. 10.1016/j.jpubeco.2020.104271 32873994 PMC7451187

[B7] Aylie N. S. Mekonen M. A. Mekuria R. M. (2020). The psychological impacts of COVID-19 pandemic among university students in Bench-Sheko Zone, South-west Ethiopia: a community-based cross-sectional study. Psychol. Res. Behav. Manag. 13, 813–821. 10.2147/PRBM.S275593 33061696 PMC7533263

[B8] Bartik A. W. Bertrand M. Cullen Z. Glaeser E. L. Luca M. Stanton C. (2020). The impact of COVID-19 on small business outcomes and expectations. Proc. Natl. Acad. Sci. U.S.A. 117, 17656–17666. 10.1073/pnas.2006991117 32651281 PMC7395529

[B9] Brunsting N. C. Zachry C. Takeuchi R. (2018). Predictors of undergraduate international student psychosocial adjustment to US universities: a systematic review from 2009-2018. Int. J. Intercult. Relat. 66, 22–33. 10.1016/j.ijintrel.2018.06.002

[B10] Cao W. Fang Z. Hou G. Han M. Xu X. Dong J.. (2020). The psychological impact of the COVID-19 epidemic on college students in China. Psychiatry Res. 287:112934. 10.1016/j.psychres.2020.112934 32229390 PMC7102633

[B11] Chen J. H. Li Y. Wu A. M. S. Tong K. K. (2020). The overlooked minority: mental health of International students worldwide under the COVID-19 pandemic and beyond. Asian J. Psychiatr. 54:102333. 10.1016/j.ajp.2020.102333 32795955 PMC7399745

[B12] Chinese Center for Disease Control Prevention. (2020). Home/COVID19. Available online at: http://www.chinacdc.cn/en/COVID19 (accessed February 01, 2020).

[B13] Collins F. E. (2021). Measuring COVID-19-related fear and threat in Australian, Indian, and Nepali university students. Pers. Individ. Dif. 175:110693. 10.1016/j.paid.2021.110693 33526955 PMC7839423

[B14] Commodari E. La Rosa V. L. (2020). Adolescents in quarantine during COVID-19 pandemic in Italy: perceived health risk, beliefs, psychological experiences and expectations for the future. Front. Psychol. 11:559951. 10.3389/fpsyg.2020.559951 33071884 PMC7538632

[B15] Conrad R. C. Hahm H. C. Koire A. Pinder-Amaker S. Liu C. H. (2021). College student mental health risks during the COVID-19 pandemic: implications of campus relocation. J. Psychiatr. Res. 36, 117–126. 10.1016/j.jpsychires.2021.01.054 33588225 PMC8635290

[B16] Fakhar-e-Alam Kulyar M. Bhutta Z. A. Shabbir S. Akhtar M. (2020). Psychosocial impact of COVID-19 outbreak on international students living in Hubei province, China. Travel Med. Infect. Dis. 37:101712. 10.1016/j.tmaid.2020.101712 32348867 PMC7194700

[B17] Flesia L. Monaro M. Mazza C. Fietta V. Colicino E. Segatto B.. (2020). Predicting perceived stress related to the COVID-19 outbreak through stable psychological traits and machine learning models. J. Clin. Med. 9:3350. 10.3390/jcm9103350 33086558 PMC7603217

[B18] Gewirtz O'Brien J. R. Auerswald C. English A. Ammerman S. Beharry M. Heerde J. A.. (2021). Youth experiencing homelessness during the COVID-19 pandemic: unique needs and practical strategies from international perspectives. J. Adolesc. Health. 68, 236–240. 10.1016/j.jadohealth.2020.11.005 33541600

[B19] Khan K. Zhao H. Zhang H. Yang H. Shah M. H. Jahanger A. (2020). The impact of COVID-19 pandemic on stock markets: an empirical analysis of world major stock indices. J. Asian Fin. Econ. Bus. 7, 463–474. 10.13106/jafeb.2020.vol7.no7.463

[B20] Lee J. Kim N. Su M. (2021). Immigrant and international college students' learning gaps: Improving academic and sociocultural readiness for career and graduate/professional education. Int. J. Educ. Res. Open 2–2:100047. 10.1016/j.ijedro.2021.100047

[B21] Liu N. Zhang F. Wei C. Jia Y. Shang Z. Sun L.. (2020). Prevalence and predictors of PTSS during COVID-19 outbreak in China hardest-hit areas: gender differences matter. Psychiatry Res. 287:112921. 10.1016/j.psychres.2020.112921 32240896 PMC7102622

[B22] Ma T. Heywood A. MacIntyre C. R. (2020). Travel health risk perceptions of Chinese international students in Australia – implications for COVID-19. Infect. Dis. Health 25, 197–204. 10.1016/j.idh.2020.03.002 32291244 PMC7128943

[B23] Ministry of Education PRC (2019). Statistical Report on International Students in China for 2018. Press Release, 3–7. Available online at: http://en.moe.gov.cn/news/press_releases/201904/t20190418_378586.html (accessed April 12, 2020).

[B24] Mirowsky J. Ross C. E. (1989). Social Causes of Psychological Distress. New York, NY: Aldine de Gruyter.

[B25] Mok K. H. Xiong W. Ke G. Cheung J. O. W. (2021). Impact of COVID-19 pandemic on international higher education and student mobility: student perspectives from mainland China and Hong Kong. Int. J. Educ. Res. 105:101718. 10.1016/j.ijer.2020.101718 PMC9188844 35719275

[B26] Nkire N. Mrklas K. Hrabok M. Gusnowski A. Vuong W. Surood S.. (2021). COVID-19 pandemic: demographic predictors of self-isolation or self-quarantine and impact of isolation and quarantine on perceived stress, anxiety, and depression. Front. Psychiatry 12:553468. 10.3389/fpsyt.2021.553468 33597900 PMC7882620

[B27] Park H.-J. Lee B. J. (2016). The role of social work for foreign residents in an epidemic: the MERS crisis in the Republic of Korea. Soc. Work Publ. Health 31, 656–664. 10.1080/19371918.2016.1160352 27351075

[B28] Quah S. R. Hin-Peng L. (2004). Crisis prevention and management during SARS outbreak, Singapore. Emerg. Infect. Dis. 10, 364–368. 10.3201/eid1002.030418 15030714 PMC3322925

[B29] Ross C. E. Mirowsky J. (1989). Explaining the social patterns of depression: control and problem solving - or support and talking. J. Health Soc. Behav. 30, 206–219. 10.2307/2137014 2738367

[B30] Sherman H. J. (1987). Foundations of Radical Political Economy. Armonk, NY: M.E. Sharpe.

[B31] Sheroun D. Wankhar D. D. Devrani A. Pv L. Gita S. Chatterjee K. (2020). A study to assess the perceived stress and coping strategies among B.Sc. nursing students of selected colleges in Pune during COVID-19 pandemic lockdown. Int. J. Sci. Healthc. Res. 5, 280–288. Available online at: https://ijshr.com/IJSHR_Vol.5_Issue.2_April2020/IJSHR_Abstract.0038.html

[B32] Stolle D. (2001). Clubs and congregations: the benefits of joining an association, in Trust in Society ed Cook K. S. (New York, NY: Russell Sage Foundation), 202–244.

[B33] Wang X. Zhang R. Wang Z. Li T. (2021). How does digital competence preserve university students' psychological well-being during the pandemic? An investigation from self-determined theory. Front. Psychol. 12:652594. 10.3389/fpsyg.2021.652594 33967915 PMC8102986

[B34] Warner-Søderholm G. Bertsch A. Sawe E. Lee D. Wolfe T. Meyer J.. (2018). Who trusts social media? Comput. Hum. Behav. 81, 303–315. 10.1016/j.chb.2017.12.026

[B35] Westhuis D. Thyer B. A. (1989). Development and validation of the Clinical Anxiety Scale: a rapid assessment instrument for empirical practice. Educ. Psychol. Meas. 49, 153–163. 10.1177/0013164489491016

[B36] World Health Organization (2021). WHO Coronavirus (COVID-19) Dashboard. Available online at: https://covid19.who.int (accessed March 02, 2021).

[B37] World Health Organization. (2020a). Coronavirus disease (COVID-19) Advice for the Public. Available online at: https://www.who.int/emergencies/diseases/novel-coronavirus-2019/advice-for-public (accessed February 01, 2020).

[B38] World Health Organization. (2020b). Coronavirus Disease (COVID-19) Q&A. Available online at:https://www.who.int/emergencies/diseases/novel-coronavirus-2019/question-and-answers-hub/q-a-detail/q-a-coronaviruses (accessed February 01, 2020).

[B39] Xiang Y.-T. Yang Y. Li W. Zhang L. Zhang Q. Cheung T.. (2020). Timely mental health care for the 2019 novel coronavirus outbreak is urgently needed. Lancet Psychiatry 7, 228–229. 10.1016/S2215-0366(20)30046-8 32032543 PMC7128153

[B40] Yan T. Zhizhong W. Jianzhong Z. Yubo Y. Jie L. Junjun Z.. (2021). Depressive and anxiety symptoms among people under quarantine during the COVID-19 epidemic in China: a cross-sectional study. Front. Psychiatry 12:566241. 10.3389/fpsyt.2021.566241 33658949 PMC7917112

[B41] Yang S. Cho S.-I. (2017). Middle East respiratory syndrome risk perception among students at a university in South Korea, 2015. Am. J. Infect. Control 45, e53–e60. 10.1016/j.ajic.2017.02.013 28385465 PMC7115287

[B42] Zeng W. Zeng Y. Xu Y. Huang D. Shao J. Wu J.. (2021). The influence of post-traumatic growth on college students' creativity during the COVID-19 pandemic: the mediating role of general self-efficacy and the moderating role of deliberate rumination. Front. Psychol. 12:665973. 10.3389/fpsyg.2021.665973 33935927 PMC8079774

[B43] Zhang K. Wu S. Xu Y. Cao W. Goetz T. Parks-Stamm E. J. (2021). Adaptability promotes student engagement under COVID-19: the multiple mediating effects of academic emotion. Front. Psychol. 11:633265. 10.3389/fpsyg.2020.633265 33488491 PMC7815756

