# Supplementary material for: Psychological Distress and Trust in University Management Among International Students During the COVID-19 Pandemic
Source: Front Psychol. 2021 Jun 18;12:679661. doi: 10.3389/fpsyg.2021.679661 (PMC8250427; doi:10.3389/fpsyg.2021.679661)
Supplement: Supplementary file 2 [file Data_Sheet_2.docx]

APPENDIX 1 – Survey items used in the study

**Psychological Distress and Trust in University Management among International Students During the COVID-19 Pandemic**

1. Your Current Residential City in China?
2. Wuhan
3. Other

1. Gender?
2. Male
3. Female
4. What is Your Current education level?
5. Undergraduate
6. Master’s
7. PhD
8. What is your age?
9. 20-29
10. 30-39
11. Marital Status
12. Single
13. Married
14. What is your nationality?
15. Asian
16. African
17. Other

**Trust in University Management**

The word **“University management”** in the following questions means the management of the university at any level, specifically the international department of the university who is responsible for the affairs of the international students in the university.

For the following questions, circle the number that you feel best describes you.

Strongly Disagree Mildly Disagree Neutral Mildly Agree Strongly Agree

1 2 3 4 5

1. University management really do care about the well -being of international students. (Benevolence 1)
2. University management really look out for what is important to me. (Benevolence 2)
3. University management is honest in their dealings with international students. (Integrity 1)
4. I always feel confident that I can rely on university management to do their part when I interact with them. (Integrity 2)
5. University management is reliable in doing what they say will be done. (Competence 1)
6. University management is effective in providing helpful advice. (Competence 2)
7. I feel connected to university management. (Identification 1)
8. University management listen to me. (Identification 2)
9. University management is sincere with their efforts to communicate with me. (Concern 1)
10. University management is concerned about my personal well-being. (Concern 2)

**Anxiety**

For the following questions, circle the number that you feel best describes you.

Strongly Disagree Mildly Disagree Neutral Mildly Agree Strongly Agree

1 2 3 4 5

1. Thinking of the way you feel these days, would you say you feel calm?
2. Thinking of the way you feel these days, would you say you feel tense?
3. Thinking of the way you feel these days, would you say you feel suddenly scared for no reason.
4. Thinking of the way you feel these days, would you say you feel nervous.
5. Thinking of the way you feel these days, would you say you feel confident about the future.
6. Thinking of the way you feel these days, would you say you have spells of terror or panic.
7. Thinking of the way you feel these days, would you say you feel nervousness or shakiness inside.
8. Thinking of the way you feel these days, would you say you feel afraid without good reason.
9. Thinking of the way you feel these days, would you say you feel generally anxious.
10. Thinking of the way you feel these days, would you say you are free from senseless or unpleasant thoughts.

**Compliance to Self-Quarantine**

If you did not develop the symptoms of Coronavirus disease (n2019) having NONCLOSE CONTACT with someone diagnosed with Coronavirus disease (n2019), Would you agree to be self-isolated for 14 days if you were asked to do so?

For the following questions, circle the number that you feel best describes you.

Strongly Disagree Mildly Disagree Neutral Mildly Agree Strongly Agree

1 2 3 4 5

**Knowledge of Disease (2019-nCov)**

1. Which one is correct about treatment or vaccine of Coronavirus (2019-nCov)?
2. There is sure treatment for Coronavirus (2019-nCoV).
3. Currently, there is neither a vaccine nor a sure Treatment for Coronavirus (2019-nCoV).
4. Currently, there isn’t a sure treatment for Coronavirus (2019-nCoV), but there is a vaccine
5. Which of the following explanations is NOT correct about the characteristics of Coronavirus (2019-nCoV)?
6. There I no scientific evidence yet that children are less susceptible to Coronavirus (2019-nCoV)
7. It is known that mortality rate is higher in elderly people who are in their 50s or above
8. There are no cases yet reported about people to people transmission
9. Which of the following is correct about the incubation period of Coronavirus (2019-nCov)?
10. Symptoms of Coronavirus (2019-nCoV) appear without an incubation period
11. During incubation period, symptoms of Coronavirus (2019-nCoV) don’t appear, but Coronavirus (2019-nCoV) can be transmitted
12. An incubation period for Coronavirus is between 2 to 7 days
13. Which of the following is correct about symptoms of Coronavirus (2019-nCoV)?
14. Typical symptoms of Coronavirus (2019-nCoV) are high fever, coughing and shortness of breath
15. Coronavirus (2019-nCoV) is not related to other underlying disease
16. Coronavirus (2019-nCoV) is cause by bacterial infection
17. Which of the following is NOT correct about “close contact” of Coronavirus (2019-nCoV)?
18. “Close contact” involves a direct contact with patient respiratory secretions
19. “Close Contact” involves being in the same room or doctor’s office, treatment room or hospital room with a Coronavirus (2019-nCoV) infected
20. Relatives and healthcare workers are excluded from the category of Coronavirus (2019-nCoV) close contact

**Preventive Measures Against Coronavirus Disease (n2019)**

1. Yes
2. No
3. In the past two weeks, if possible I did not leave home/room/dormitory
4. In the past two weeks, I wash my hands with water and soap before and after I leave home/room/dormitory
5. In the past two weeks, I don’t touch my eyes nose, and/or mouth with hands I have not washed
6. In the past two weeks, I avoid contact with people who have fever or respiratory symptoms
7. In the past two weeks, I wash my hands with hand sanitizers when I came from outside
8. In the past two weeks, I wear a mask when I go outside
9. In the past two weeks, I avoid crowded places
10. In the past two weeks, I go hospital much less
11. In the past two weeks, I avoid using public transportation
12. In the past two weeks, I eat food/medicine that would strengthen my immune system
13. In the past two weeks, I do physical exercise to strengthen my immune system

**Self-Health Perception**

1. Yes
2. No

In the past two weeks, have you had any of the following symptoms?

1. Dry Cough
2. Flu
3. Shortness of Breath
4. Persistent high fever of 38°C (100.4°F) or higher, lasting for a day or more
5. Sore Throat
6. Having aches all over the body
